# ‘Your hopes can run away with your realistic expectations’: a qualitative study of women and men’s decision-making when undergoing multiple cycles of IVF

**DOI:** 10.1093/hropen/hoaa059

**Published:** 2020-12-23

**Authors:** T Copp, D Kvesic, D Lieberman, D Bateson, K J McCaffery

**Affiliations:** 1 Sydney School of Public Health, Faculty of Medicine and Health, The University of Sydney, Sydney, Australia; 2 Sydney Health Literacy Lab, Sydney School of Public Health, Faculty of Medicine and Health, The University of Sydney, Sydney, Australia; 3 City Fertility Centre, Sydney, Australia; 4 Family Planning NSW, Ashfield, NSW, Australia; 5 Discipline of Obstetrics, Gynaecology and Neonatology, Faculty of Medicine and Health, The University of Sydney, Sydney, Australia

**Keywords:** IVF, multiple cycles, decision-making, psychological wellbeing, fertility

## Abstract

**STUDY QUESTION:**

What are the factors that contribute to the decision to continue or stop IVF treatment after multiple unsuccessful cycles?

**SUMMARY ANSWER:**

Factors contributing to the decision included external factors, such as their doctor’s guidance, success rates, the outcomes of previous cycles and anecdotal stories of success, as well as emotional and cognitive drivers, including perception of success, hope and fear of regret.

**WHAT IS KNOWN ALREADY:**

Infertility affects about one in six Australian couples of reproductive age. Regarding IVF, some couples with a good prognosis drop out of treatment prematurely, whilst others continue for multiple cycles, despite limited chances of success. Little is known about what factors contribute to the decision to continue IVF after multiple failed cycles.

**STUDY DESIGN, SIZE, DURATION:**

Semi-structured face-to-face and telephone interviews were conducted with 22 participants. Interviews were audio-recorded, transcribed and analysed thematically using Framework analysis.

**PARTICIPANTS/MATERIALS, SETTING, METHODS:**

Doctors and nurses at an Australian private fertility clinic recruited individuals and/or couples who had undergone three or more complete unsuccessful cycles of IVF.

**MAIN RESULTS AND THE ROLE OF CHANCE:**

The majority of participants had decided to or were leaning towards continuing treatment. Participants expressed a range of common factors important in their decision-making, which were evident both within and across couples. For most, their doctor’s advice and hope were key factors influencing their decision. Most participants expressed they would continue as long as there was a chance of success and until their doctor advised otherwise. Other factors included participants’ perception of their likelihood of success, hearing anecdotal stories of success after multiple cycles, positive outcomes of previous cycles and fear of regret.

**LIMITATIONS, REASONS FOR CAUTION:**

The sample was highly educated and recruited from one private Australian fertility clinic only. Many participants were also couples, which may have resulted in more homogenous data as they shared the same diagnosis for infertility and outcomes of previous cycles. Factors influencing the decision to continue or stop may differ in different sociodemographic populations and in other healthcare systems.

**WIDER IMPLICATIONS OF THE FINDINGS:**

Given the important role of the doctor’s guidance and patients’ own perceptions of their likelihood of success, which they tended to overestimate, it is vital that fertility specialists give accurate and transparent information regarding their likelihood of success and continue to regularly communicate this throughout the IVF journey. Anecdotal stories of success against the odds appeared to be influential in the decision to continue and underpinned unrealistic perceptions of possible success. More personalized, cumulative estimates of likelihood of success may help couples with their decision-making as well as with discussions about ending treatment or setting a limit before commencing IVF.

**STUDY FUNDING/COMPETING INTEREST(S):**

The study was funded by the National Health and Medical Research Council (NHMRC) Program Grant (APP1113532). No further competing interests exist.

**WHAT DOES THIS MEAN FOR PATIENTS?**

Deciding whether to continue or stop IVF after several unsuccessful rounds is difficult and little is known about women and men’s decision-making on this issue.

This study explored the decision to continue or stop IVF after multiple cycles. Key factors for continuing described by women and men included their doctor’s advice and still feeling hopeful that they would be successful. Other factors influencing the decision included positive perceptions of their chance of success and fear of regret. Further factors included positive outcomes of previous IVF cycles (good number of eggs, achieving a pregnancy) and hearing stories of success despite very slim chances.

These findings highlight the important role of the doctor in providing accurate and objective information to patients about their chances of success. Regular discussions and counselling about stopping may help manage unrealistic expectations.

## Introduction

Infertility affects about one in six couples of reproductive age worldwide (Evers, 2002; Herbert *et al.*, 2009). Infertility is unexpected and shocking and can be an extremely stressful experience (Cousineau and Domar, 2007), with most people wanting and expecting to have children at some stage in their life (Holton *et al.*, 2011; Peterson *et al.*, 2012). Although infertility rates have remained stable, an increasing number of couples are seeking specialist care (Evers, 2002). There are now a number of available assisted reproductive treatments (ARTs), such as IVF, which have transformed the treatment of infertility (Chambers *et al.*, 2017). National estimates indicate 4.4% of all women who gave birth in Australia in 2015 received some form of ART (AIHW, 2016). IVF is the most used technique and its use has grown immensely in high-income countries (Te Velde *et al.*, 2017). Although treatment can bring renewed hope, IVF is also new source of stress, as it is physically demanding, emotionally taxing, financially costly and time-consuming (Peddie *et al.*, 2005; Peterson *et al.*, 2006).

A treatment cycle of IVF typically consists of ovarian stimulation, the retrieval of oocytes, fertilization of the collected oocytes and embryo culture, then one or more resulting embryos are transferred into to the woman’s uterus in a fresh transfer, and/or surplus embryos placed in cryostorage (Fitzgerald *et al.*, 2018). An IVF cycle is complete when all fresh and frozen embryos have been transferred. Despite its growing use and advances, success rates of IVF remain modest. In 2016, 18.1% of all initiated autologous cycles in Australia and New Zealand (76 225 recorded) resulted in a live delivery (Fitzgerald *et al.*, 2018). Research, however, has found the public holds unrealistic expectations about IVF success rates, often expecting immediate success (Wyndham *et al.*, 2012; Hammarberg, 2017; Prior *et al.*, 2019).

The optimal number (most cost effective and clinically effective) of IVF cycles is typically considered to be three complete cycles (National Institute for Health and Care Excellence, 2013). However, a number of patients with a good prognosis of achieving a pregnancy drop out of treatment prematurely (e.g. after one cycle), whist others continue for several cycles, despite a low likelihood of success (Callan *et al.*, 1988; Klock, 2015). In 2016, 10.7% of women undergoing IVF in Australia had four or more autologous cycles (Fitzgerald *et al.*, 2018). Cumulative success rates of IVF show little chance of pregnancy after approximately the fifth cycle of treatment, regardless of the woman’s age (Chambers *et al.*, 2017). Despite this, the decision to continue or stop treatment after multiple IVF failures is challenging, and couples are often unsure whether they should continue or stop treatment (Braverman, 1997; Peddie *et al.*, 2005; Klock, 2015). Given the substantial physical, psychological and financial burden of IVF, concerns have been raised about the potential harms of unrealistic expectations and patients continuing to pursue treatment indefinitely (Annual Capri Workshop Group, 2019). Infertile couples are vulnerable and obscured by their strong desire of parenthood, putting them at high risk of overdiagnosis and overtreatment (Annual Capri Workshop Group, 2019). There may also be commercial drivers at play (e.g. financial conflicts of interest) encouraging an overly positive viewpoint on the chances of success (Blakely *et al.*, 2019).

A number of studies have investigated why patients stop or drop out of treatment, with the most common factors including psychological distress, financial costs, disruption to lives and careers, physical impact, relationship strain and having to travel long distances for treatment (Peddie *et al.*, 2005; Gameiro*et al.*, 2012). However, there is a paucity of published data on the factors that contribute to patients pursuing multiple cycles of IVF despite limited chance of success (Callan *et al.*, 1988; Mesquita da Silva *et al.*, 2018). Differences in how men and women cope with IVF (e.g. women rely more on social support, confrontative coping and accepting responsibility, whilst men used more distancing and planful problem-solving strategies) have been identified (Peterson *et al.*, 2006), as well as differences in their involvement in treatment decision-making (Mesquita da Silva *et al.*, 2018), highlighting the importance of capturing both men and women’s perspectives. This study aimed to explore the psychological and external factors that contribute to the decision to continue or stop IVF treatment for both women and men.

## Materials and methods

### Design

Using semi-structured face-to-face and telephone interviews, this qualitative study explored women and men’s experiences of undergoing multiple cycles of IVF, and the psychological and external factors that influence the decision-making process (see Supplementary Table S1 for the Consolidated Criteria for Reporting Qualitative Studies checklist).

### Ethics approval

Study methods were approved by The University of Sydney Human Research Ethics Committee (2017/743) and Genea Ethics Committee (GEC0031).

### Participants and recruitment

Participants were patients who had undergone three or more complete cycles of IVF. Three complete cycles were chosen as it is typically considered the optimal number (most cost effective and clinically effective) of cycles to undertake (National Institute for Health and Care Excellence, 2013), with diminishing benefit in success rates beyond three cycles (Chambers *et al.*, 2017). Eligible patients and/or couples were given a recruitment package by their doctor or nurse, which contained a letter of invitation, the participant information statement, an expression of interest (EOI) form and reply-paid envelope. Interested participants completed the EOI form and returned it to the researchers at the University of Sydney. Participants were then contacted to organize a time for the interview and provide written informed consent. Although both members of the couple were invited, it was not a requirement that both participated. Recruitment continued until preliminary analysis indicated thematic consistency regarding factors influencing the decision to continue, indicating saturation of key themes (Braun and Clarke, 2013).

### Data collection

The semi-structured interview schedule was developed by the research team and informed by themes identified in previous IVF literature (Hall and Lieberman, 2016). A fertility counsellor reviewed the interview schedule and it was piloted with one woman who had undergone IVF to ensure all questions were appropriately framed and sensitive. Topics included the impact of IVF, experience of undergoing multiple cycles, the decision to stop or continue treatment and the factors contributing to the decision. This article focuses on the data from the latter two topics (Supplementary Table S2). Interviews were conducted face-to-face or by phone by T.C. and D.K., depending on the participants’ preference (most chose by phone, n = 20), between January 2018 and October 2019. They lasted between 30 and 100 min (median = 46 min) and were audio-recorded and transcribed verbatim. Although both members of the couple were invited to participate, participants were interviewed separately as this study was focused on individual perceptions, experiences and decision-making, not interpersonal or collective shared decision-making. In addition, whilst there are benefits to joint interviewing (e.g. it saves time and enables dyadic analysis i.e. observation of the couple’s dynamics, interaction, shared experience and meanings; Taylor and de Vocht, 2011), there is evidence that joint interviewing can generate relationship tension and prevent researchers from giving an equal voice to both partners, resulting in a one-sided narrative (Taylor and de Vocht, 2011; Zarhin, 2018).

### Analysis

To better understand individual perceptions, experiences and decision-making, we used a phenomenological qualitative approach, which is concerned with understanding people’s subjective experiences (Braun and Clarke, 2013). Data were analysed thematically using Framework analysis, which uses a matrix-based approach, where columns depict themes and rows list participants, enabling the relationships between the themes and participants to be explored (Ritchie and Spencer, 1994; Ritchie*et al.*, 2003). Two researchers (T.C. and D.K.) reviewed the transcripts and developed a list of emerging topics and salient themes, which were discussed in-depth with K.J.M. and formed the basis of the coding framework. The interviews were then coded into the framework by D.K. and T.C., with iterations to the framework made as required through continuous discussion with K.J.M. A random subset (15%) was double coded to ensure consistency, with any differences discussed and reassessed. Prominent themes were then discussed with the research team and examined within and across couples to develop the interpretation of the results.

## Results

The 22 participants included 13 women and 9 men, of which there were 9 dyads (both members of the couple were interviewed). The mean age for both women and men was 39 years. The majority of participants had a university degree (77%) and were trying for their first child (59%). The mean number of completed IVF cycles for their current attempt of conceiving was 4.6. All participants were in heterosexual relationships and undertaking IVF with their partner. Of the 22 participants, 12 were leaning towards continuing IVF (including 5 couples), 2 were unsure (1 couple), 6 were leaning towards stopping (2 couples) and 2 were considering continuing with donor oocytes (1 couple) (Table I).

**Table I:** Demographic and treatment-related characteristics of the sample (n = 22).^*^

	Females (n = 13)	Males (n = 9)
Current age (years)		
30–34	3	2
35–39	4	3
40–44	6	3
45+	0	1
Education		
HSC (Year 12)	1	1
Diploma/trade certificate	2	1
Bachelor degree or above	10	7
Number of children		
0 (i.e. trying for first child)	8	5
1	5	4
Complete cycles of IVF		
3–5	11	7
6–8	2	2
Leaning towards continuing or stopping IVF?		
Continuing	6	4
Unsure	1	1
Stopping	5	3
Continuing with donor oocytes	1	1

* n = 22 in total, including 9 dyads (both members of the couple participated in the study).

**Figure 1.**
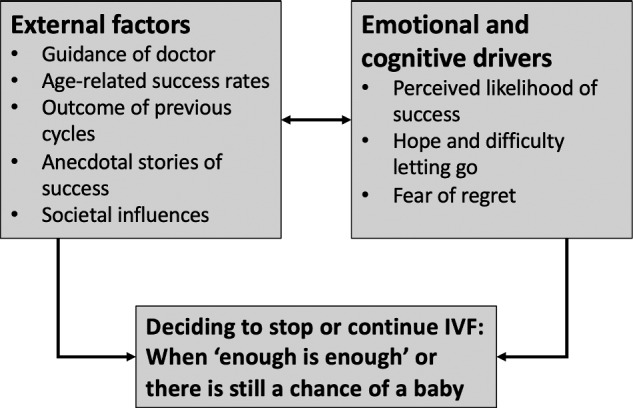
Overview of overarching themes and subthemes regarding the factors influencing the decision to continue or stop IVF treatment.

The key factors influencing the decision-making process varied widely both within and across couples and were organized into two overarching areas: (i) external factors, including their doctor’s guidance, age-related success rates, the outcomes of previous cycles, anecdotal stories of success and societal influences, and (ii) emotional and cognitive drivers, including perceptions of success, hope, optimism and fear of regret (see Fig. 1). The language used to describe the results reflects both the frequency and the extent that themes were emphasized within and across participants. Participant quotes are also included to illustrate themes.

### External factors

#### Guidance of doctor

The doctor was reported to be an important figure for almost all participants in guiding the IVF process, and many described how they value and rely on their doctor’s expertise and knowledge to provide statistics around their chance of success, to inform about possible options, as well as to reassess options after each cycle. Most participants reported they will continue with IVF unless their doctor advises otherwise.

‘*I am someone who believes and respects authority a lot, so if she says there's a chance, then there's a chance*’. (Female, 35–39 years, 3 complete cycles)

One participant reported this was the case even if they personally felt ambivalent about continuing.

‘*When you see a doctor that does give you hope you’re sort of forced into, you’re emotionally forced into doing another cycle*’. (Male, 30–34 years, 5 complete cycles)

#### Age-related success rates

Many participants described how their age and the corresponding statistics of success are important factors influencing their decision to continue or stop IVF. For participants in their 30s, their younger age caused them to feel positive about their chances, and they described feeling that if they keep trying, they will eventually be successful.

‘*I've only just turned 32. I think the advice that we’ve been given by most of the specialists that your chances of this working before you hit 35 are really high. It’s just a numbers game. Our intention now would be not to stop until I get to an age where the fertility specialists say, look, the chances are pretty slim*’. (Female, 30–34 years, 5 complete cycles)

On the other hand, participants in their late 30s or early 40s described how they feel time is running out, so need to fit in as many cycles as possible before they get older.

#### Outcome of previous cycles

Positive outcomes of previous cycles were also described as a factor influencing the decision to continue. For example, some participants often discussed how previous positive outcomes, such as a good number of oocytes collected or achieving a biochemical pregnancy, provided the perception of progressing forward, generating hope that the following cycles would be successful.

‘*…if I wasn’t getting good eggs and stuff or the egg numbers dropped off, then basically the deal is we would be stopping a whole lot sooner*’. (Female, 40–44 years, 4 complete cycles)

In addition, participants who reached pregnancy described feeling that, despite a failed pregnancy, achieving a pregnancy means that the IVF process is working and they may succeed if they continue.

‘*I may do another one because we still got a good result to a degree, whereas if we hadn’t got that positive, I would’ve said no, we’re done*’. (Female, 40–44 years, 4 complete cycles)

#### Anecdotal stories of success

Some participants mentioned anecdotal stories of success in achieving a live birth through IVF when discussing their decision to continue. Participants reported hearing these anecdotes from a range of sources, including their doctor, friends, family members and online. Anecdotal stories of success against the odds seemed to play a subtle but important role in their perceptions of success and their decision to continue, contributing to their sense of hope and increasing uncertainty about when to stop.

‘*I know there’s one woman from this clinic who was successful on her 12th cycle…it makes you think, when do you stop then?*’ (Female, 40–44 years, 4 complete cycles)

#### Societal influences

Despite most participants stating that their social relationships had no direct impact on their decision-making, a few described how receiving advice and support (emotional or financial) from friends and family could subtly affect their decision to continue.

‘*My partner’s mum would say like, oh, you’ve got to keep trying. You know, it only takes one*’. (Female, 30–34 years, 5 complete cycles)

A few participants also mentioned social pressures to have children, and how this could indirectly influence their decision.

‘*I mean it might impact our decision somewhat because all our friends have kids and it might spur you on more to try another cycle when maybe you wouldn’t have in the past*’. (Male, 35–39 years, 7 complete cycles)

### Emotional and cognitive drivers

#### Perceived likelihood of success

The way participants perceived their chances of success also seemed to be important in influencing their decision. Whilst participants unanimously reported holding unrealistic expectations when first starting IVF, most described becoming aware that the chances of success are low, recalling realistic statistics for their age. However, those who wished to continue still perceived this small chance in a positive light as ‘*it wasn’t a zero percent chance*’ (Male, 45–49 years, 4 complete cycles), and described feeling optimistic and hopeful that they would achieve a successful outcome.

‘*I want to remain optimistic because scientifically I know that there is a chance that it can happen. Um, but I obviously know that the odds are quite low. But because there is a chance that it can happen there’s no reason to think that it won’t happen*’. (Female, 40–44 years, 5 complete cycles)

Some participants described the process of IVF in gambling terms when discussing their likelihood of success, and how IVF is ‘*like winning the lottery ticket*’. One participant, who has taken a break from IVF, likened IVF to be like an ‘*expensive gambling habit*’ which felt a ‘*tiny bit addictive*’ in creating the mindset of ‘*let’s go again*’ when the previous cycle was unsuccessful (Female, 35–39 years, 4 complete cycles). Others also reported recalling being told by their clinician that the IVF process is like a ‘*numbers game, the more cycles you have, ultimately you will get there*’.

#### Hope and difficulty letting go

Hope appeared to play an important role for many participants and influenced many of the factors described above. Many participants described feeling unwilling to stop treatment as they want to give it their best shot, even if they had already passed their previously decided upon number of attempts. This illustrates the strong emotional drive underpinning the decision to continue, and the difficulty in letting go of their dream of having their own biological child.

‘*I can feel myself getting into that mode of, oh no, I don’t want to stop yet. I guess that’s something I wasn’t prepared for, and thinking, oh just try one more*’. (Female, 40–44 years, 4 complete cycles)

Some participants also described how stopping IVF ‘*would be a really difficult decision*’, as stopping would mean that ‘*you know that you’re not going to have a baby*’ (Female, 40–44 years, 5 complete cycles). They described feeling unwilling to give up on IVF as they perceived it as their best chance of having a baby.

‘*I’m leaning probably now to, although I said I wouldn’t do it, doing another round, because if I don’t then I’m giving up, and I don’t want to give up*’. (Female, 40–44 years, 4 complete cycles)

Others stated that they have not even considered stopping IVF and described feeling unwilling to set a limit on the number of cycles as they are determined to do everything they can.

‘*We were told by one of the specialists, you guys need to put a number on this, and we went away and spoke about it and neither of us were really willing to do that*’. (Female, 30–34 years, 5 complete cycles)

#### Fear of regret

Many participants described feeling they need to continue with IVF to do everything in their power to have a baby in order to avoid any sense of regret later in life.

‘S*o we don’t want in ten years’ time to look back and say, why didn’t we do more cycles? So there’s that worry that we’ll regret our decision*’. (Male, 30–34 years, 5 complete cycles)

This was the case even if they described believing they will not be successful.

‘*I honestly feel like I'm doing this next cycle purely to avoid an emotional or psychological issue later in my life when I look back and think I didn’t do enough. I don’t believe for one minute we're going to fall pregnant on these next two cycles*’. (Female, 35–39 years, 3 complete cycles)

### Analysis within couples

As the data include perspectives from both members of the same couple, there is some overlap with regards to the treatment-related characteristics (infertility diagnosis, number of cycles) and factors influencing decision-making (doctor’s advice, outcomes of previous cycles). Whilst there were indeed many similarities regarding the factors raised by each member of the couple, analysis within dyads also identified several differences in salient factors within couples, that also varied substantially across couples, with all themes evident both within and across dyads (see Supplementary Table S3). A couple of gender differences in decision-making were identified; men often described feeling willing to continue with IVF for as long as their partners wished to do so, whereas women tended to describe more social pressures to continue IVF, more often described being unwilling to stop and also discussed the physical impact of treatment.

‘*I don’t want to deny her the chance to have her own biological baby. I basically said that I can’t make the final decision. I’m willing to keep going as long as you are*’. (Male, 30–34 years, 5 complete cycles)

The decision to continue was mostly described by both members of the dyads as mainly the woman’s decision, with the main reason being that they are the ones who physically go through treatment.

‘*He wasn’t the decision maker. I was the decision maker, because it’s my body*’. (Female, 40–44 years, 3 complete cycles)

Some, however, described it as ‘*a shared decision*’, and that ‘*it was important that we both were happy with the decision that we made*’ (Female, 40–44 years, 4 complete cycles). Only one female participant said that her partner was driving the decision to continue as ‘*he’s probably more wanting to have a second child*’. (Female, 40–44 years, 4 complete cycles)

### When enough is enough: deciding to stop IVF

Six of the 22 participants (including two dyads) had decided to stop IVF at the time of the interview. The main reasons for stopping included their doctor’s guidance and the psychological, physical and financial impact of IVF. These participants described how IVF has had a significant impact on their emotional and psychological wellbeing, the IVF process making them feel ‘*like a science project*’ and ‘*emotionally numb*’. They described their desire to move on with life and focus on what they have already.

‘*I didn’t want to have a baby at all costs, at the cost of me and my health, and my relationship with my partner*’. (Female, 35–39 years, 4 complete cycles)

Despite the many reported challenges of IVF, participants described the decision to stop as extremely difficult, with a few finding it easier to consider themselves as taking a break from IVF rather than stopping for good, illustrating the difficulty in letting go of their dream of having their own biological child.

‘*I’m saying no now, but I’m not saying I won’t ever do it again. It’s just something that I just need to have a break from for now*’. (Female, 35–39 years, 4 complete cycles)

A few participants described the difficulty in shifting their mindset from feeling unrealistically optimistic to being more realistic about the likelihood of success. This further illustrates how ‘*your hopes can run away with your realistic expectations*’. (Male, 45–49 years, 4 complete cycles)

‘*It’s how to balance having realistic thinking as well. So not searching constantly for the one percent. How to shift your thinking around that at some point. That is what we found hard*’. (Female, 40–44 years, 4 complete cycles)

Prominently, these participants described how their doctor played an important role in helping them come to their decision, providing them with realistic information about their chances of success and the outcomes of their previous cycles, as well as counselling about stopping.

‘*I think seeing the specialist last week, who pretty much said, you’ve given it a fair go, you’ve put everything into it and the results aren’t very positive. It would be reasonable to finish up now. So giving that advice was, I think, the thing that has probably made the final decision for us*’. (Male, 30–34 years, 5 complete cycles)

## Discussion

Although decisions were highly individualized and influenced by personal circumstances, participants expressed a range of factors important in their decision-making about continuing or stopping IVF after multiple failed attempts, which were evident both within and across couples. Key factors included their doctor’s guidance, hope, perceptions of success, anecdotal stories of success after multiple attempts, positive outcomes of previous cycles and fear of regret.

While many described being aware that their chance of success was minimal, those wishing to continue often still interpreted their likelihood of success in a positive light, illustrating their optimism. These findings align with a number of known cognitive influences and biases that affect decision-making, such as confirmation bias (the tendency to search for, interpret and recall information in a way that confirms or strengthens one’s prior personal beliefs; Plous, 1993) or optimism bias (where one believes that they themselves are less likely to experience a negative event; Sharot, 2011). Similar to findings in both patient and public populations (Hammarberg *et al.*, 2001; Peddie *et al.*, 2005; Wyndham *et al.*, 2012; Fauser*et al.*, 2019 ), all participants in the current study reported holding unrealistic expectations going into IVF, expecting IVF to be a quick fix. Many described how with each cycle, they came to increasingly recognize the possibility of treatment failure, although most of those continuing remained hopeful that they would be successful. These findings build on previous quantitative research, where although the psychological burden of treatment was the same for those who continued with treatment versus those who discontinued, those who continued were more optimistic about the chance of success with further attempts (Callan *et al.*, 1988). This illustrates their unwillingness to let go of their biological parenthood goal (Mesquita da Silva *et al.*, 2018) and highlights their increased vulnerability to holding unrealistic expectations, increasing the risk of over-persistence and overtreatment (Annual Capri Workshop Group, 2019).

Importantly, these findings underscore the vital need for doctors to repeatedly give accurate, upfront and transparent information regarding patients’ likelihood of success throughout the IVF journey to manage unrealistic expectations (Mesquita da Silva *et al.*, 2018). This may help reduce the risk of over-persistence and the associated psychological, physical and financial costs (Annual Capri Workshop Group, 2019). Whilst difficult, thorough discussions about the possibility of failure may help in enabling realistic expectations from the beginning (Klock, 2015), enabling clinicians to maintain positive relationships with patients whilst being transparent about risks, benefits and alternatives (Ethics Committee of the American Society for Reproductive Medicine, 2012). Participants also expressed a clear need for personally relevant statistics for their specific situation (e.g. age, diagnosis, previous IVF outcomes), as identified in previous research (Mesquita da Silva *et al.*, 2018). More specific, easily accessible information may help patients with their decision-making. Anecdotal stories of success also seemed to play an influential role in fuelling hope. For example, hearing of friends or women at the same clinic having success after a large number of cycles contributed to unrealistic expectations of IVF effectiveness. When people are trying to conceive, success stories are often publicized, whilst negative examples are often not shared socially (Hammarberg *et al.*, 2001; Kelly-Hedrick *et al.*, 2018), making success appear more common and achievable when in fact it is rare (availability bias; Tversky and Kahneman, 1973). Studies have also shown that people tend to reject evidence-based information in favour of anecdotal information, highlighting their undue influence on medical decision-making (Ubel *et al.*, 2001; Jaramillo*et al.*, 2019). Taken together with previous research, these findings emphasize the importance for clinicians to use great caution when discussing success stories against the odds. Balancing them against anecdotes of failure or pairing them with easily understandable statistical evidence, such as using graphical formats (Fagerlin *et al.*, 2005), may reduce reliance on anecdotes of success in decision-making. Furthermore, clinicians need to be mindful when expressing optimism which, in both this study and in other quantitative research, was found to influence patients’ decision to continue, even if patients themselves are ambivalent (Callan *et al.*, 1988).

Those leaning towards stopping treatment described how their doctor’s advice and counselling about stopping was extremely influential in coming to terms with failure, which in addition to experiencing poor outcomes of previous cycles, contributed to feeling that continuing would be futile and letting go of hope. Other reasons for stopping after three or more cycles are similar to those found in previous literature (Gameiro *et al.*, 2012), where physical, emotional and financial exhaustion have been found to be why couples discontinue treatment. These findings illustrate that hope plays an instrumental role in the decision to end treatment after multiple cycles, which is ultimately bound up in the person’s ability to let go of the dream of having their own biological child (Braverman, 1997; Mesquita da Silva *et al.*, 2018). It is important to note that a few participants said it was psychologically important to continue, despite believing success would be unlikely. Patients can perceive benefit differently to clinicians, and continuing can help them feel they have tried all efforts to reproduce, even if unsuccessful (Hammarberg *et al.*, 2001; Ethics Committee of the American Society for Reproductive Medicine, 2012). This highlights that continuing can bring psychological benefit for some patients, even if chances are minimal. Although counselling is widely available in Australia (through IVF clinics and general practitioners), only a few participants in the current study sought psychological support. However, those who did described it as helpful in reaching the decision to stop. Finding ways to increase uptake of psychological support services may assist patients when facing this difficult decision.

Strengths of this study include the recruitment of both women and men, and the participants being interviewed during or just after the decision-making process, minimizing recall bias. The interview schedule was also developed by a multidisciplinary team, and rigorous qualitative methods were used in analysis to reach final themes. Limitations include the highly educated and homogenous nature of the sample who were recruited from one private Australian clinic only. Many participants were also couples, which may have resulted in more homogenous data as they shared the same diagnosis for infertility and outcomes of previous cycles. Recruitment was also very challenging, as this can be a very sensitive and confronting topic to discuss (Peddie *et al.*, 2005). Although recruitment of both men and women is a strength, couples were recruited together, meaning that if men were in strong disagreement with their partner, they may have been unlikely to participate. In addition, most participants were interviewed over the phone. Although we believed that their partner was not present during the interview, this was not checked so it is possible that the partner was present, which could have potentially influenced their answers. However, we did not record any background sound or comments during any of our interviews which would indicate another person is present. Factors influencing the decision to continue may differ for men recruited without their partners, homosexual couples or single women going through IVF independently, and also may differ in different sociodemographic populations and in other healthcare systems. Further qualitative and quantitative research in larger samples from more diverse cultural, social and economic backgrounds is warranted.

In conclusion, these findings provide valuable insights into the decision-making process for women and men undergoing multiple cycles of IVF. These findings highlight the crucial role of the doctor in providing objective, realistic guidance and in providing this information repeatedly to couples as they continue their IVF journey. There may also be a role for a decision tool to support individuals and couples when facing this difficult decision (Mesquita da Silva *et al.*, 2018). Along with more personally relevant statistics, this could facilitate regular communication about the possibility of treatment being unsuccessful, helping patients accept that being unsuccessful is a realistic possibility.

## Supplementary data

Supplementary data are available at *Human Reproduction Open* online.

## Supplementary Material

hoaa059_Supplementary_Data
