# Supplementary material for: ‘Your hopes can run away with your realistic expectations’: a qualitative study of women and men’s decision-making when undergoing multiple cycles of IVF
Source: Hum Reprod Open. 2020 Dec 23;2020(4):hoaa059. doi: 10.1093/hropen/hoaa059 (PMC7757429; doi:10.1093/hropen/hoaa059)
Supplement: hoaa059_Supplementary_Data [file hoaa059_supplementary_data.zip › HRO-20-0047-R2-SuppTable2.docx]

**Supplementary Table 2. A qualitative study of women and men’s decision-making when undergoing multiple cycles of in-vitro fertilisation: Interview schedule**

**INTRODUCTION**

Thank you for agreeing to be interviewed. As you know, we are doing a qualitative study to explore women’s and couples’ experiences with continuing or stopping IVF treatment. So, in this interview we are interested in your own experiences with IVF, the factors involved in your decision-making about IVF, and how it may have impacted your life.

There are no right or wrong answers; it is really to help me understand your thoughts, feelings and experiences, so please feel free to say anything about what it has been like for you, and you don’t have to answer any question that you don’t want to.

This interview will be audio-recorded to make sure we have an accurate record of your responses, but everything you say will be kept strictly confidential. This is a safe space, nothing you say will affect your treatment at [fertility clinic] or your relationship with your doctor.

So, if that is alright with you I will start the audio-recording now, okay? Do you have any questions before we begin?

**STORY OF GETTING TO IVF, INFERTILITY AND TREATMENT HISTORY**

**So, we are interviewing women and families with a variety of experiences, some who have children already but are undergoing IVF for further children, and some who are trying for their first child. Can you tell me where you sit, do you have any children?**

**Some people go through IVF independently or with their partners, can you please tell me what your circumstances are?**

**Can you please tell me about your story on how you came to seek IVF?**

**How many complete stimulated treatment cycles of IVF you have had to date?** (complete cycle meaning a whole cycle from hormone stimulation, egg collection and the use of all collected fresh and frozen embryos)

**When you first started IVF, did you set an expectation of how many cycles you would have?**

**IVF EXPERIENCE**

**Can you describe what impact IVF has had on your life, in either a positive or negative way?**

*Prompt:* Has IVF impacted your physical health, work, life opportunities, finances, lifestyle, relationships, social life, faith in any way?

**Has IVF impacted your psychological wellbeing in either a positive or negative way?**

**What emotions do you experience when going through IVF? How has IVF impacted your wellbeing, in either a positive or negative way?**

**EXPERIENCE OF MULTIPLE ROUNDS**

**So you’ve been through a few rounds of IVF now, does the impact of each round vary? If so, how does it vary?**

**STOPPING OR CONTINUING TREATMENT**

**Thinking about the future, do you intend to have another IVF attempt/ cycle within the next 6 or 12 months?**

***If stopping:* Would you try again at some point in the future, or have decided to stop for good?**

***If continuing:* Have you thought about how many more cycles you may have?**

**FACTORS CONTRIBUTING TO DECISION MAKING**

**What factors will contribute/ have contributed to your decision about whether to stop or continue treatment?**

*Prompt* (**stopping treatment**): Could you please describe to me how you came to the final decision to stop IVF?

*Prompt* (**continuing treatment**): Could you please describe to me what factors helped you decide to have another round?

*Prompt* (**unsure**): For you, what are the pros and cons to stop or continue treatment? What factors will contribute to your decision?

**What role does/did your partner play in making this decision?**

**What are your expectations about undergoing another cycle?**

**Does your fertility specialist participate in your decision? / provide any guidance or advice?**

**You mentioned the impact of IVF on your financial situation/ work/ career/ holidays/ social life/ lifestyle/ physical health/ impact on relationship, how much does that play a role in your decision?**

**Do/did your family or friends have any role in the decision-making process?**

**Have you ever sought counselling support for your IVF journey?**

*Prompt:* Has this played a role in the decision-making process?

**QUALITY OF DECISION MADE**

**Do you feel clear about what is important to you in your decision to stop/ continue treatment?**

**We are interested in the process that people go through to make the decision about continuing or deciding to stop that is right for them. How do you feel about your decision to continue/stop treatment?**

*Prompt:* Are you satisfied with the process you went through with the decision you have made?

**Is there any information that you would have liked to have known that you weren’t told about?**

**Have you ever considered going to another clinic?**

**Have you thought about any other alternatives beyond IVF?**

**Is there anything else that you would like to add in relation to your thoughts, experiences and feelings?**

**DEMOGRAPHICS**

**Age**

**Education**

**Occupation**
