# Supplementary material for: ‘Your hopes can run away with your realistic expectations’: a qualitative study of women and men’s decision-making when undergoing multiple cycles of IVF
Source: Hum Reprod Open. 2020 Dec 23;2020(4):hoaa059. doi: 10.1093/hropen/hoaa059 (PMC7757429; doi:10.1093/hropen/hoaa059)
Supplement: hoaa059_Supplementary_Data [file hoaa059_supplementary_data.zip › HRO-20-0047-R3-SuppTable1.docx]

**Supplementary Table 1. COREQ checklist: A qualitative study of women and men’s decision-making when undergoing multiple cycles of in-vitro fertilisation**

**The Consolidated Criteria for Reporting Qualitative Studies (COREQ): 32-item checklist**

(Table adapted from Tong et al., 2007)

| **No. Item** | **Guide questions/description** | **Notes** |
| --- | --- | --- |
| **Domain 1: Research team and reﬂexivity** | | |
| **The research team**  The multidisciplinary research team had expertise in qualitative methodology (TC, KM, DK), psychology (KM, TC), medical decision-making (KM), obstetrics and gynaecology (DL, DB), and assisted reproductive technologies (DL). | | |
| ***Personal characteristics*** | |  |
| 1. Interviewer/  facilitator | Which author/s conducted the interview or focus group? | Interviews were conducted by TC and DK |
| 2. Credentials | What were the researcher’s credentials? E.g. PhD, MD | TC: BA (Hons in Psychology), PhD  DK: BA |
| 3. Occupation | What was their occupation at the time of the study? | At the time of the interviews, TC was a PhD candidate and DK was a research assistant |
| 4. Gender | Was the researcher male or female? | The interviewers (TC and DK) were female |
| 5. Experience and training | What experience or training did the researcher(s) have? | TC and DK were trained in qualitative methods |
| ***Relationship with participants*** | | |
| 6. Relationship established | Was a relationship established prior to study commencement? | TC and DK did not have any contact with participants prior to organising the time for the interview |
| 7. Participant knowledge of the interviewer | What did the participants know about the researcher? e.g. personal goals, reasons for doing the research | Participants were informed that researchers at the University of Sydney were interested in exploring decision making around undergoing multiple cycles of IVF |
| 8. Interviewer characteristics | What characteristics were reported about the inter viewer/facilitator? e.g. Bias, assumptions, reasons and interests in the research topic | Participants were informed that the researchers were interested in exploring decision making around undergoing multiple cycles of IVF, with the ultimate aim of designing a decision tool to help couples when facing this difficult decision |
| **Domain 2: Study design** | | |
| ***Theoretical framework* (see Methods section of paper for more detail)** | | |
| 9. Methodological orientation and Theory | What methodological orientation was stated to underpin the study? e.g. grounded theory, discourse analysis, ethnography, phenomenology, content analysis | Methods in this study were based on phenomenology and framework analysis. As phenomenological methodology focuses on individual experience, this theoretical orientation was appropriate to explore the experience of undergoing multiple cycles of IVF |
| ***Participant selection* (see Methods)** | | |
| 10. Sampling | How were participants selected? e.g. purposive, convenience, consecutive, snowball | Participants were a convenience sample recruited through a private IVF clinic in Sydney, Australia |
| 11. Method of approach | How were participants approached? e.g. face-to-face, telephone, mail, email | Clinicians gave eligible patients a recruitment pack, containing information about the study and expression of interest form |
| 12. Sample size | How many participants were in the study? | There were 22 participants in the study |
| 13. Non-participation | How many people refused to participate or dropped out? Reasons? | There were no participants who dropped out after consenting to participate. It is unknown how many participants were given information about the study and did not participate. |
| ***Setting* (see Methods)** | | |
| 14. Setting of data collection | Where was the data collected? e.g. home, clinic, workplace | Interviews were conducted over the phone or face-to-face at The University of Sydney, as preferred by the participant |
| 15. Presence of non-participants | Was anyone else present besides the participants and researchers? | Only the participant and researcher were present at the time of the interview |
| 16. Description of sample | What are the important characteristics of the sample? e.g. demographic data, date | Interviews were conducted between January 2018 and October 2019. See Results and Table I for demographics |
| ***Data collection* (see Methods)** | | |
| 17. Interview guide | Were questions, prompts, guides provided by the authors? Was it pilot tested? | Interviews were semi-structured and roughly followed the interview schedule in Supplementary Data B. The interview schedule was pilot tested with 1 woman who had undergone IVF and was also reviewed by a fertility counsellor |
| 18. Repeat interviews | Were repeat inter views carried out? If yes, how many? | No repeat interviews were carried out |
| 19. Audio/visual recording | Did the research use audio or visual recording to collect the data? | All interviews were audio-recorded |
| 20. Field notes | Were ﬁeld notes made during and/or after the interview or focus group? | Field notes were made throughout the interviews. Relevant themes were identified during preliminary analysis to help inform the analysis framework |
| 21. Duration | What was the duration of the interviews or focus group? | Interviews lasted between 30-120 minutes |
| 22. Data saturation | Was data saturation discussed? | Preliminary analysis during data collection suggested thematic consistency regarding factors influencing the decision to continue. |
| 23. Transcripts returned | Were transcripts returned to participants for comment and/or correction? | Transcripts were not returned to participants for comment and/or correction |
| **Domain 3: Analysis and findings** | | |
| ***Data analysis* (see Methods)** | | |
| 24. Number of data coders | How many data coders coded the data? | DK and TC coded the entire dataset, with a subset (15%) double coded. |
| 25. Description of the coding tree | Did authors provide a description of the coding tree? | Themes and subthemes are described at the beginning of the Results. |
| 26. Derivation of themes | Were themes identified in advance or derived from the data? | Themes were derived from the data |
| 27. Software | What software, if applicable, was used to manage the data? | Microsoft Excel was used for Framework analysis |
| 28. Participant checking | Did participants provide feedback on the findings? | No |
| ***Reporting* (see Results)** | | |
| 29. Quotations presented | Were participant quotations presented to illustrate the themes/ findings? Was each quotation identified? E.g. participant number | Participant quotes from various participants are presented to illustrate themes. All quotes are identified with participant ID, sex, age and number of complete cycles |
| 30. Data and findings consistent | Was there consistency between the data presented and the findings? | The quotes presented are consistent with the findings described |
| 31. Clarity of major themes | Were major themes clearly presented in the findings? | Major themes are presented under subheadings |
| 32. Clarity of minor themes | Is there a description of diverse cases or discussion of minor themes? | Diverse cases and minor subthemes are included |
