# Supplementary material for: ‘Your hopes can run away with your realistic expectations’: a qualitative study of women and men’s decision-making when undergoing multiple cycles of IVF
Source: Hum Reprod Open. 2020 Dec 23;2020(4):hoaa059. doi: 10.1093/hropen/hoaa059 (PMC7757429; doi:10.1093/hropen/hoaa059)
Supplement: hoaa059_Supplementary_Data [file hoaa059_supplementary_data.zip › HRO-20-0047-R3-SuppTable3.docx]

| **Couple/ Individual**  **Supplementary Table 3. Main themes by couple or individual** | **Leaning towards continuing or stopping** | **Cycles of IVF** | **Sex (F, M), age** | **Factors influencing the decision** |
| --- | --- | --- | --- | --- |
| **Data grouped by couple (both members of the couple participated)** | | | | |
| Couple 1 | Continue | 3 | F, 35-39 years | External: **age-related success rates**, outcomes of previous cycles, guidance of doctor, anecdotal stories of success**, societal influences**  Emotional and cognitive: **fear of regret, difficulty letting go** |
|  |  |  | M, 40-44 years | External: outcome of previous cycles, guidance of doctor, anecdotal stories of success  Emotional and cognitive: **supporting partner** |
| Couple 2 | Continue | 5 | F, 30-34 years | External: age-related success rates, guidance of doctor  Emotional and cognitive: **difficulty letting go, perceived likelihood of success** |
|  |  |  | M, 35-39 years | External: age-related success rates, guidance of doctor, **outcomes of previous cycles, anecdotal stories of success**  Emotional and cognitive: **fear of regret, supporting partner** |
| Couple 3 | Unsure | 4 | F, 40-44 years | External: guidance of doctor, **outcomes of previous cycles, societal influences**  Emotional and cognitive: fear of regret, hope, difficulty letting go, wants to give daughter a sibling |
|  |  |  | M, 35-39 years | External: guidance of doctor, **anecdotal stories of success**  Emotional and cognitive: hope, difficulty letting go, fear of regret, wants to give daughter a sibling, but **also wants to get life back** |
| Couple 4 | Continue | 4 | F, 40-44 years | External: **outcomes of previous cycles**, guidance of doctor, **anecdotal stories of success, societal influences**  Emotional and cognitive: Hope, perceived likelihood of success, **difficulty letting go** |
|  |  |  | M, 30-34 years | External: **age-related success rates**, guidance of doctor  Emotional and cognitive: hope, perceived likelihood of success, **big toll of IVF on partner** |
| Couple 5 | Continue | 7 | F, 35-39 years | External: age-related success rates, guidance of doctor, outcomes of previous cycles  Emotional and cognitive: **hope, perceived likelihood of success,** **difficulty letting go** |
|  |  |  | M, 35-39 years | External: age-related success rates, outcomes of previous cycles, guidance of doctor  Emotional and cognitive: **supporting partner**, **societal influences** |
| Couple 6 | Stopping | 4 | F, 40-44 years | External: poor outcomes of previous cycles, age-related success rates, guidance of doctor, **concerns about risks of age on pregnancy**  Emotional and cognitive: emotional toll, lost hope, desire to get life back |
|  |  |  | M, 45+ years | External: age-related success rates, outcomes of previous cycles, guidance of doctor  Emotional and cognitive: financial and emotional toll, desire to get life back vs **fear of regret,** gave it best shot, lost hope |
| Couple 7 | Continue with donor | 3 | F, 40-44 years | External: **guidance of doctor**, poor outcomes of previous cycles, age-related success rates, higher chances with donor eggs  Emotional and cognitive: hope, didn’t want to wait any longer |
|  |  |  | M, 40-44 years | External: poor outcomes of previous cycles, age-related success rates, higher chances with donor eggs  Emotional and cognitive: **supporting partner,** hope |
| Couple 8 | Continue | 7 | F, 40-44 years | External: age-related success rates, guidance of doctor  Emotional and cognitive: difficulty letting go, although financial and relationship toll, hope, perceived likelihood of success |
|  |  |  | M, 40-44 years | External: age-related success rates, guidance of doctor, **outcomes of previous cycles**  Emotional and cognitive: hope, perceived likelihood of success, trust in IVF process, **societal influences** |
| Couple 9 | Stopping | 5 | F, 30-34 years | External: guidance of doctor, **physical impact, poor outcome of previous** **cycles**  Emotional and cognitive: lost hope (feels futile), desire to get life back |
|  |  |  | M, 30-34 years | External: guidance of doctor  Emotional and cognitive**: impact on partner, supporting partner**, hope and **fear of regret** |
| **Individuals (partner did not participate in an interview)** | | | | |
| Individual 1 | Stopping | 4 | F, 35-39 years | External: age-related success rates, poor outcomes of previous cycles, guidance of doctor  Emotional and cognitive: loss of hope, wants to get life back, big emotional and financial impact |
| Individual 2 | Continue | 4 | F, 40-44 years | External: Outcomes of previous cycles, age-related success rates  Emotional and cognitive: wants to give it best shot, societal influences |
| Individual 3 | Continue | 4 | F, 40-44 years | External: age-related success rates, guidance of doctor, anecdotal stories of success  Emotional and cognitive: hope, perceived likelihood of success, difficulty letting go |
| Individual 4 | Stopping | 5 | F, 35-39 years | External: poor outcomes of previous cycles, ran out of sperm, big physical impact of IVF, societal influences  Emotional and cognitive: financial and emotional toll, desire to get life back, doesn’t want a baby at all costs |

***Bold indicates differences within couples**
